# Comparative machine learning survival models for under-five mortality in Southern Africa with frailty modelling

**DOI:** 10.1016/j.dialog.2026.100326

**Published:** 2026-07-27

**Authors:** Mafanedza Nephawe, Wende Clarence Safari, Pierre-Philippe Dechant, Justine B. Nasejje

**Affiliations:** a School of Computer Science & Applied Mathematics, University of Witwatersrand, Johannesburg, South Africa; b Inequalities in Cancer Outcomes Network (ICON), Department of Health Research Services and Policy (HSRP), London School of Hygiene & Tropical Medicine, London, United Kingdom; c School of Mathematics, University of Leeds, Leeds, United Kingdom; d School of Statistics and Actuarial Science, University of Witwatersrand, Johannesburg, South Africa

**Keywords:** DeepFrailty, DeepHit, Random survival forest, Child survival, Variable importance, Clusters, Demographic health survey

## Abstract

**Purpose:**

Child survival remains central to achieving Sustainable Development Goals (SDGs) 3 and 10, particularly in sub-Saharan Africa where inequalities in health outcomes persist. This study aimed to predict under-five survival in Southern Africa using advanced machine learning survival models and to examine the influence of household composition, maternal care giving, and unobserved community-level heterogeneity on child mortality outcomes.

**Methods:**

A retrospective cohort was constructed using Demographic and Health Survey (DHS) birth-history data from Malawi, South Africa, Zambia, and Zimbabwe. Survival time was measured from birth until death before age five or censoring at the time of interview, with a maximum follow-up period of 59 months. Three survival modelling approaches were evaluated: Random Survival Forests (RSF), DeepHit, and DeepFrailty. The DeepFrailty model incorporated DHS cluster identifiers to account for unobserved community-level heterogeneity. Model performance was assessed using the concordance index (C-index) and Integrated Brier Score (IBS), while calibration analyses and five-fold cross-validation were used to evaluate predictive reliability and model robustness.

**Results:**

Survival probabilities remained consistently high across all four countries, exceeding 0.97 throughout the follow-up period. South Africa generally demonstrated the most favourable survival outcomes and lowest mortality risk, whereas Zambia showed comparatively lower survival probabilities and higher cumulative hazard levels over time. Among the evaluated models, RSF achieved the strongest predictive performance across countries, with C-index values ranging from 0.8890 to 0.9458 and IBS values ranging from 0.0062 to 0.0097. DeepHit demonstrated slightly lower predictive performance (C-index: 0.5889–0.8943; IBS: 0.0085–0.0140), while DeepFrailty produced comparatively lower predictive accuracy (C-index: 0.8385–0.9159; IBS: 0.0164–0.0212). However, DeepFrailty provided clearer separation of survival and cumulative hazard curves across countries by capturing latent community-level heterogeneity through the frailty component. Important predictors of under-five survival included “births in the past year”, “total number of living children”, “total children ever born”, “currently breastfeeding”, and “birth order”, highlighting the importance of household composition, fertility behaviour, and maternal caregiving practices in child survival.

**Conclusion:**

The findings demonstrate the potential of machine learning survival models for improving under-five mortality prediction and risk stratification in Southern Africa. RSF emerged as the most accurate and robust predictive model, while DeepFrailty provided valuable insights into unobserved socioeconomic and community-level heterogeneity influencing child survival. The results further emphasise the importance of targeted, context-specific interventions focusing on maternal healthcare, family planning, breastfeeding promotion, and household-level support to improve child survival outcomes. Future work should focus on improving the interpretability of deep learning survival models and incorporating additional contextual information to support evidence-based public health interventions aligned with SDGs 3 and 10.

## Highlights

• Under-five survival exceeded 97% across Malawi, South Africa, Zambia, and Zimbabwe.
• Random Survival Forest achieved the highest predictive accuracy among all models tested.
• DeepFrailty revealed hidden community-level heterogeneity shaping child mortality risk.
• Breastfeeding, birth order, and fertility history strongly predicted under-five survival.
• Findings support targeted maternal and household interventions aligned with SDGs 3 and 10.

## 1 Introduction

Under-five child mortality remains a major global health challenge, particularly in sub-Saharan Africa (sSA) [1]. Effective interventions require high-quality data to guide targeted strategies, in line with the Sustainable Development Goals (SDGs) [2]. Achieving SDG 3 (Good Health and Well-being) and SDG 10 (reducing inequalities) depends on analysing survival outcomes across individual, household, and community levels [3], enabling identification of vulnerable populations and health systems with unmet needs.

Children within the same household or community may have correlated survival outcomes due to shared environmental, social, or healthcare factors. Traditional survival methods, such as random survival forests (RSF), can estimate survival probabilities while relaxing parametric assumptions but do not explicitly account for clustering [4]. Deep learning approaches, including DeepHit and DeepFrailty, provide flexible frameworks for survival modelling. DeepFrailty, in particular, incorporates frailty terms to capture clustering effects, accounting for unobserved heterogeneity and nonlinear relationships among predictors.

By integrating hierarchical structure with neural network flexibility, DeepFrailty uncovers complex patterns and interactions that influence under-five survival across households and communities, providing interpretable predictions for population-level risk assessment [5], [6], [7], [8], [9].

This study applies the DeepFrailty model to predict under-five survival in Southern Africa using data from Malawi, South Africa, Zambia, and Zimbabwe. Its novelty lies in combining multilevel, clustered data with deep learning to capture both observed and unobserved heterogeneity, improving understanding of child survival determinants and supporting evidence-based interventions.

## 2 Data source and methods

### 2.1 DHS datasets

We constructed a retrospective survival cohort from cross-sectional DHS birth history data. DHS surveys follow a standard two-stage cluster sampling design: enumeration areas are first selected from national census data, and households are systematically sampled within these clusters. Eligible women (aged 15– 49) provide retrospective birth histories, along with household, maternal, and child-level information [10]. These data enable quantification of survival outcomes for children under five.

The DHS datasets are publicly available, nationally representative, and provide detailed birth histories, allowing estimation of child mortality hazards and survival probabilities [10]. They also support rigorous analysis of survival in settings where mortality is often clustered within households or communities [11], [12], [13].

**Fig. 1:**
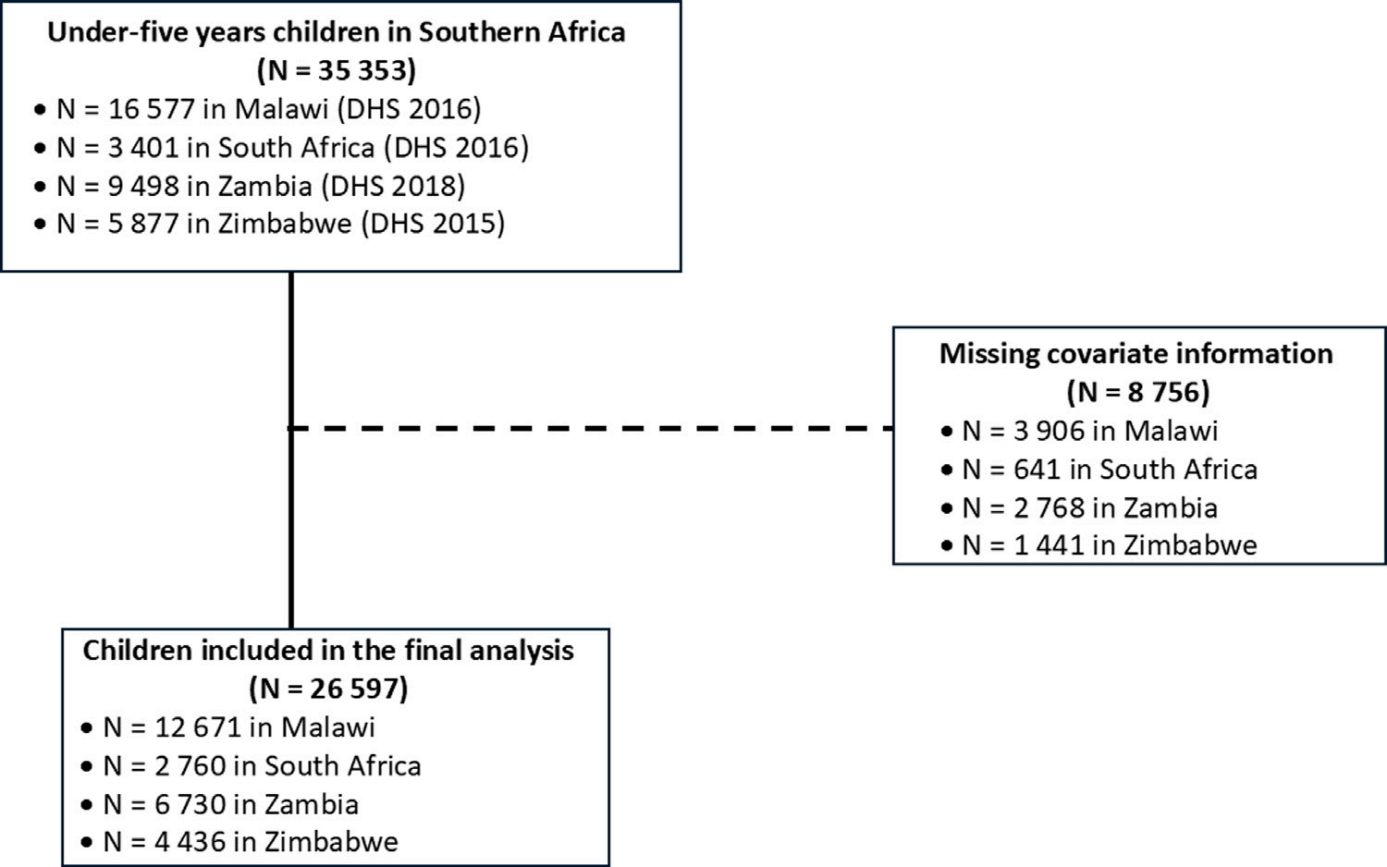
Flow diagram of under-five children in Malawi, South Africa, Zambia, and Zimbabwe based on DHS survey data.

We used the most recent DHS surveys: Malawi (2016), South Africa (2016), Zambia (2018), and Zimbabwe (2015). We included singleton live births within five years prior to the survey. We excluded children with missing age at death or survival status, survival times over 59 months, or missing covariate data that could not be harmonised across countries. We started with 35,353 live births and our final analytic cohort included 26,597 children: 12,671 from Malawi, 2760 from South Africa, 6730 from Zambia, and 4436 from Zimbabwe (Fig. 1).

### 2.2 Methods

#### 2.2.1 Nature of survival data

We reconstructed individual time-to-event data from reported birth histories. Let T denote the survival time from birth until death before the age of five years or censoring, and let $\delta_{i}$ represent the event indicator for child i, where $\delta_{i}=1$ if the child died before age five and $\delta_{i}=0$ if the child was still alive at the time of the survey or had not experienced the event by the end of follow-up. Thus, children who remained alive at the time of the survey were treated as right-censored observations at their recorded follow-up time. Survival time was measured in months, with a maximum follow-up period of 59 months [4].

#### 2.2.2 Methodological background

Survival analysis quantifies the probability that a child will survive beyond a specified time t [14], is expressed as

$$S\left(t|x\right)=\mathrm{Pr}\left(T>t|\mathrm{X = x}\right),\;$$

where, T a positive random variable, represents the time from birth to death or censorship (end of follow-up) at the end of the study. The values of S(t∣x) range between 0 and 1 and exhibit a monotonically decreasing relationship with t. At the start of follow-up t=0, none of the children have died, so the survival probability S(t=0∣x)=1. The hazard function, $\lambda \left(t|x\right)$, on the other hand, models the conditional failure rate. It is interpreted as the conditional probability of the event occurring in a very small time interval of size, $\left(\delta t\right)$ following time, t as δt approaches zero [14]. It is given as

$$\lambda \left(t|x\right)=\underset{\delta t\to 0}{\lim}\frac{\mathrm{Pr}\left(t\leq T\leq t+\delta t|T\geq t,\mathrm{X = x}\right)}{\delta t}.$$

The cumulative hazard function Λ(t∣x) is deduced by calculating the integral of the hazard function from 0 to t [14] as

$$\Lambda \left(t|x\right)=\int_{0}^{t}\lambda \left(u|x\right)\delta u.$$

The terms “variables” and “covariates” will be used interchangeably and denoted as X = x.

The random survival forest is formed by using a bootstrapping process, where B datasets are sampled from the original dataset, and B trees are subsequently grown, as mentioned in [14]. Each tree yields a cumulative hazard, $\Lambda \left(t|x\right)$, and a survival function, $S\left(t|x\right)=\exp\left(-\Lambda \left(t|x\right)\right)$, can be derived thereafter.

The random survival forest method produces the cumulative hazard function for the entire forest as its result by aggregating the cumulative hazard functions computed by each individual tree and then dividing this sum by the total number of trees, denoted as B, as described in [14].

$$\Lambda \left(t|x\right)=\frac{1}{B}\overset{B}{\underset{b=1}{\sum }}\Lambda_{b}\left(t|x\right).$$

In DeepHit, time is treated discretely, and the time variable T is partitioned into intervals of equal duration $\tau_{0},\tau_{1},\ldots ,\tau_{n}$ before being entered into the network for a given set of covariates X = x as input [15]. The final layer of a DeepHit model for a single event is triggered by a softmax activation function, resulting in a sequence of anticipated likelihoods $y\left(x\right)$ that is computed for time instances 0 through n. This vector is represented as $y\left(x\right)={\left[y_{0}\left(x\right),y_{1}\left(x\right),\ldots ,y_{n}\left(x\right)\right]}^{T}$ [15].

A survival function in time $\tau_{i}$ for covariates X = x can be represented as;

(1) S^τi∣x=1−∑k=1Jykx.

The objective function, denoted by $L_{tot}$ that is minimised during the training of the DeepHit model is obtained by summing up two distinct losses: $L_{1}$ and $L_{2}$ [15].

The $L_{1}$ loss function is designed to handle right-censored data and consists of two components. The first term, $\delta_{i}\log\left(y_{ei}\left(x_{i}\right)\right)$, provides information about uncensored instances i that experience the event e under consideration. The second term, 1−δilogS^Ti∣xi, pertains to individuals in the dataset who have encountered censoring. This $L_{1}$ loss is derived by computing the negative sum from one to the total count of individuals N, encompassing both censored and uncensored observations within the dataset, as described in [15] and [16]. The representation of the $L_{1}$ loss function is therefore given as

(2) L1=−∑i=1Nδilogyeixi+1−δilogS^Ti∣xi.

On the other hand, the $L_{2}$ ranking loss function incorporates the concept of concordance, which ensures that a patient who dies at time t is assigned a higher risk at time t than a patient who survived longer than t. Therefore for pairs $\left(i,j\right)$ where the risks can be compared (i.e., concordant pairs), the loss function penalises incorrect predictions made by the DeepHit model. It achieves this by incorporating the difference between the estimated survival probabilities of i and j S^Ti∣xi−S^Ti∣xj, divided by a penalizer term σ. The result is then exponentiated. $L_{2}$ is multiplied by a hyperparameter α [14], which determines its weight relative to the total DeepHit loss $\left(L_{tot}=L_{1}+L_{2}\right)$ [14]. Therefore, $L_{2}$ can be formally expressed as;

(3) L2=α∑i≠jδi1{Ti≤Tj}exp−S^Ti∣xi−S^Ti∣xjσ.

In this case, the hyperparameters α that denote the relative importance of the ranking loss and the penalizer σ need to be optimised [16]. Consider a positive scalar random variable $\nu \in R^{+}$ that expresses the unobserved heterogeneity corresponding to individuals, or frailty. The conditional hazard function in the presence of frailty is defined as [17], [18]

$$\lambda \left(t|x,\nu \right)=\nu \lambda \left(t|x\right).$$

The frailty variable, ν, specific to each individual, is integrated into the hazard function. Additionally, the distribution of ν can be described using a one-dimensional parameter, denoted as θ∈R. The NFM [17] is a neural approximation to the logarithm of the conditional hazard function

$$\log\lambda \left(t|x,\nu \right)=\log\left(\nu \right)+\log\left(\lambda \left(t|x\right)\right)=\text{NN}\left(x,t,\log\left(\nu \right);\;\theta \right)\;.$$

Here NN represents the neural network that takes covariates X = x, time t, the logarithm of the frailty variable $\log\left(\nu \right)$, and parameters θ, to produce the logarithm of the hazard function, incorporating both the effects of covariates and the frailty term. Mathematically, the C-index can be defined as;

$$C=\frac{\text{Number of Concordant Pairs}}{\text{Total Number of Pairs}}\;.$$

In the case of survival analysis, the C-index can be further extended to include censored observations [19]. Let $T_{i}$ be the true survival time for the individual i, $T_{j}$ be the true survival time for subject j, $\delta_{i}$ be the censored indicator for individual i, and 1(⋅), indicator function that equals 1 if the condition is true and 0 otherwise. The C-index with censoring is given by;

C=∑i<jδiδj1Ti<Tj×1S^Ti>S^Tj+121Ti=Tj×1S^Ti=S^Tj∑i<jδiδj.

Here, S^t represents the estimated survival function from the survival model. The Brier score is defined as;

BS(t∣x)=1N∑i=1N1(ti≤t∧δi=1)0−S^(t∣xi)2G^(ti∣x)+1(ti>t)1−S^(t∣xi)2G^(t∣x),

where $t_{i}$ is the observed time for individual i, S^(t∣xi), the predicted survival probability for individual i at time t, G^(t∣x), the estimated survival probability from the censoring distribution.

To ensure robust model development and internal validation while maximising use of the available data, all models were trained and evaluated using 5-fold cross-validation, with an 80/20 split between training and validation data in each fold. Categorical variables were transformed using one-hot encoding, resulting in high-dimensional sparse feature representations for the DeepHit and DeepFrailty models. Continuous variables were standardised prior to model training where applicable. To prevent data leakage, preprocessing procedures, including encoding and scaling, were performed separately within each training fold and subsequently applied to the corresponding validation fold during cross-validation. All analyses were performed using the Python programming language (version 3.9) [20]. The models were implemented using PyTorch (version 1.13) and the pycox library (version 0.2.3), with torchtuples (version 0.2.2) used for model training and optimisation. Data preprocessing and statistical analyses were conducted using NumPy (version 1.23), pandas (version 1.5), and scikit-learn (version 1.2). All figures and visualisations were generated using Matplotlib (version 3.6).

### 2.3 Study variables

We reconstructed individual time-to-event data from reported birth histories. Time zero was the child’s date of birth. Children were followed until death before age five (event) or until age five or the survey date (censoring). The maximum follow-up was 59 months.

Key predictors included:

• **Individual-level:** child’s sex.
• **Household-level:** mother’s age at first birth, parental education, birth order, births in the past five years, total children born, number of living children, maternal marital status, wealth status, religion, postpartum amenorrhoea, current pregnancy, breastfeeding.
• **Community and health system-level:** drinking water source and delivery care.

### 2.4 Random survival forest

We used the RSF to model under-five survival, relaxing the proportional hazards assumption of Cox models. RSF builds an ensemble of decision trees using the log-rank test to split nodes. Cumulative hazards are estimated in each terminal node using the Nelson-Aalen estimator and aggregated across trees to obtain ensemble cumulative hazards or survival probabilities. Survival curves are derived via the Kaplan–Meier estimator. Each terminal node contains a minimum number of events to ensure reliable estimates [14].

We assessed predictor importance using Permutation Feature Importance (PFI) [21]. PFI measures the decrease in the model’s C-index when each feature is randomly permuted, providing an interpretable ranking of predictors.

**Fig. 2:**
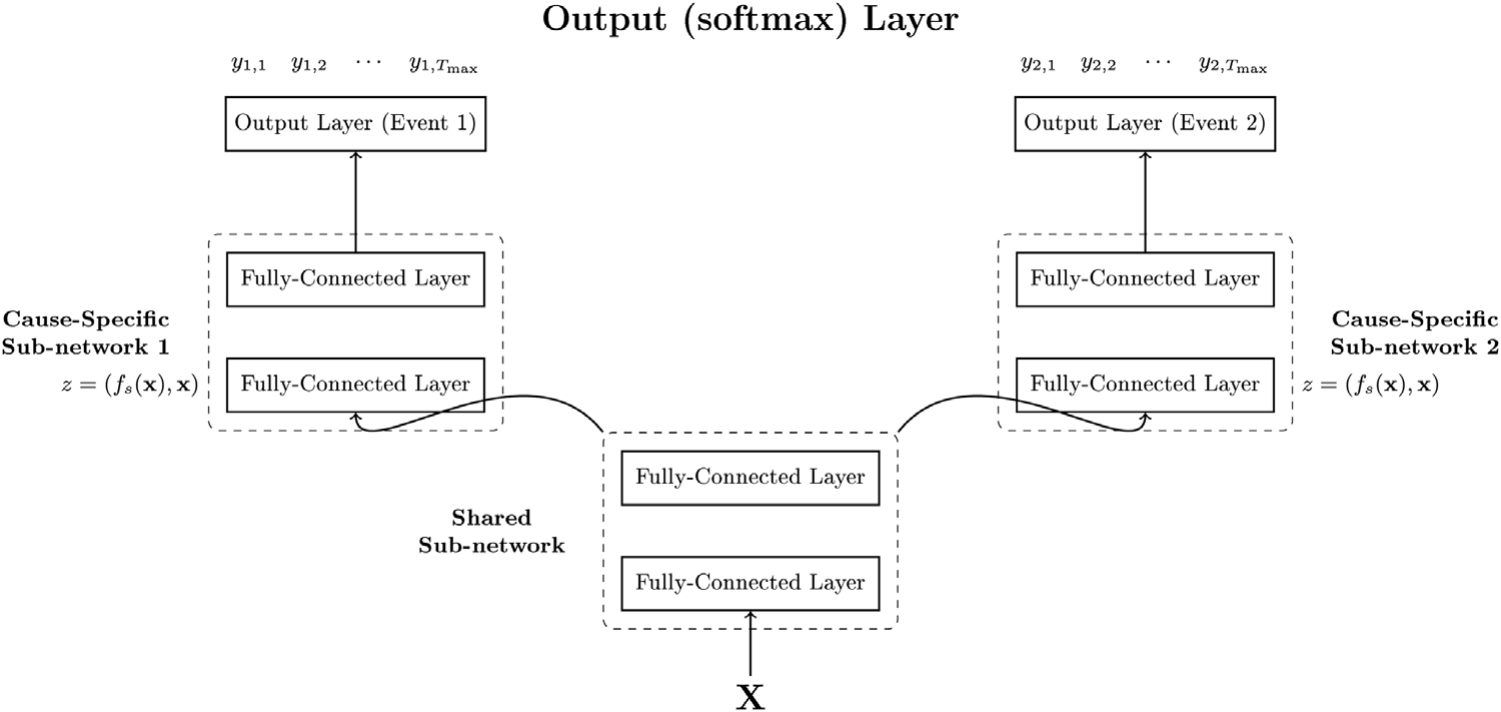
Feed-forward neural network architecture of the DeepHit model used for survival analysis [15].

### 2.5 Deep learning survival methods

Deep learning models have advanced survival analysis by capturing complex, non-linear relationships among covariates [15]. Extensions of Cox models, such as DeepSurv [22], and RNN-based parametric models (e.g., RNN-SURV [23], Weibull RNN allow flexible time-to-event predictions [24].) DeepHit accommodates single-event and competing-risk survival data without relying on proportional hazards assumptions [15].

#### 2.5.1 DeepHit

DeepHit uses a feed-forward neural network to predict discrete survival probabilities over time (Fig. 2). Unlike Cox and DeepSurv models, DeepHit handles non-proportional hazards and correlated events. Its fully connected layers use ReLU activations and a softmax output to estimate survival probabilities at discrete time intervals [15]. We implemented DeepHit using the Pycox package, constructing two hidden layers with dropout for regularisation. The output maps to a discretised time grid, allowing the model to learn survival probabilities across intervals. We applied early stopping during training and reported survival curves for under-five mortality across the four countries (Table 1).

**Table 1:** Hyperparameters for DeepHit and DeepFrailty models used in this study.

Hyperparameter	DeepHit	DeepFrailty
Input Features	924	621 (one-hot encoded categorical)
Architecture	MLP [32,32] plus output layer	MLP [128,1]
Activation Function	ReLU	ReLU
Dropout Rate	0.1	0.5
Number of Events	1	1
Output	Discrete-time survival	log-risk score
Monotonic Layer Hidden Units	–	128
Frailty Term	–	Integrated Generalized Gamma (IGG)
Positive Transformation	–	Exponential
Optimiser	Adam	Adam
Learning Rate	0.01	0.001
Loss Function	DeepHit loss (likelihood and ranking)	Monotone Negative Log-Likelihood
Alpha (likelihood weight)	0.2	–
Sigma (ranking loss bandwidth)	0.1	–
Weight Decay	0	0.01
Batch Size	64	256
Epochs	20	30
Cross-validation	5-fold CV split and seed 42	5-fold CV split and seed 77
Evaluation Metrics	C-index, IBS	C-index, IBS

### 2.6 Neural frailty machine or DeepFrailty

The machine learning and deep learning models we have explored so far do not incorporate the concept of frailty. The Neural Frailty Machine (NFM) framework adopts the classical concept of multiplicative frailty from survival analysis within the proportional hazards framework, which is used to account for unobserved differences among individuals [25]. The NFM framework incorporates unobserved heterogeneity into neural survival regression models, including many commonly used survival regression models as special cases. This model was adopted in this study to account for the unobserved heterogeneity, which is very common in datasets on children health.

We used a neural network model, we call DeepFrailty, to predict the mortality risk of under-fives using DHS data. The DeepFrailty model consists of a feedforward neural network with two hidden layers, the first maps the input to 128 hidden units using a ReLU activation and dropout regularisation, and the second projects to a scalar log-risk score. A frailty component is captured through a monotonic transformation layer based on the Integrated Generalized Gamma (IGG) distribution, which ensures the output is a valid survival function. The model was trained using the Monotone Negative Log-Likelihood loss, optimised with the Adam algorithm. Five-fold cross-validation was used, with model selection based on the minimum validation loss. Evaluation was conducted using the concordance index (C-index) and Integrated Brier Score (IBS) (Table 1).

The datasets were preprocessed to include 26 variables, such as maternal age group, source of drinking water, birth order, education levels, and household characteristics. These covariates were one-hot encoded, resulting in a total of 924 input features. Missing values were excluded from the analysis. The outcome variable was time to event (in months), where the event was defined as child death before the age of five. Censoring was indicated by a status variable.

### 2.7 Measures of model performance

#### 2.7.1 C-index

In the field of survival analysis, the concordance index (C-index), also referred to as the concordance probability [26], is the most commonly employed metric for evaluating the predictive capabilities of survival models. This metric measures the model’s capacity to accurately rank pairs of survival times for distinct subjects. A higher C-index indicates better predictive accuracy [27].

The C-index is computed by evaluating the fraction of concordant pairs among all pairs of subjects. Two subjects are considered concordant if the one who experiences the event of interest earlier also has a higher predicted risk compared to the one who experiences the event later. The C-index has a scale ranging from 0.5, suggesting random prediction to 1.0 which indicates a perfect prediction.

#### 2.7.2 Brier score

It is used to measure the overall performance of the survival model. The advantage of the Brier score is that it is a proper scoring rule that addresses both calibration and discrimination [28]. It is calculated as the mean squared difference between the observed survival status and the predicted survival probability. Specifically, at a given time point t, the Brier score for an individual is determined by squaring the difference between the observed event outcome and the model’s predicted probability of survival up to t. A lower Brier score indicates a well-calibrated and predictive model.

**Table 2:** Characteristics of children from Malawi, South Africa, Zambia and Zimbabwe.

Characteristic	Malawi	South Africa	Zambia	Zimbabwe
	n/N(%)	n/N(%)	n/N(%)	n/N(%)
**Sex of child**				
Male	8293/16 577 (50.0)	1752/3401 (51.5)	4705/9498 (49.5)	2889/5877 (49.2)
Female	8284/16 577 (50.0)	1649/3401 (48.5)	4793/9498 (50.5)	2988/5877 (50.8)
**Child vital status**				
Alive	16 211/16 577 (97.8)	3348/3401 (98.4)	9292/9498 (97.8)	5713/5877 (97.2)
Dead	366/16 577 (2.2)	53/3401 (1.6)	206/9498 (2.2)	164/5877 (2.8)
**Mother’s age at first birth**				
15−19 years	11 554/16 577 (69.7)	1573/3401 (46.3)	6582/9498 (69.3)	3322/5877 (56.5)
20−29 years	4916/16 577 (29.7)	1720/3401 (50.6)	2829/9498 (29.8)	2481/5877 (42.2)
30−45 years	107/16 577 (0.6)	108/3401 (3.2)	87/9498 (0.9)	74/5877 (1.3)
**Mother’s education**				
None	11 501/16 577 (69.4)	242/3401 (7.1)	4483/9498 (47.2)	758/5877 (12.9)
Complete primary	3937/16 577 (23.7)	1885/3401 (55.4)	3999/9498 (42.1)	4688/5877 (79.8)
Complete secondary	861/16 577 (5.2)	935/3401 (27.5)	627/9498 (6.6)	75/5877 (1.3)
Higher education	278/16 577 (1.7)	339/3401 (10.0)	389/9498 (4.1)	356/5877 (6.1)
**Father’s education**				
None	7047/13666 (51.6)	169/1362 (12.4)	2042/7057 (28.9)	397/4937 (8.0)
Complete primary	4081/13666 (29.9)	563/1362 (41.3)	3446/7057 (48.8)	3856/4937 (78.1)
Complete secondary	1923/13666 (14.1)	458/1362 (33.6)	999/7057 (14.2)	202/4937 (4.1)
Higher education	615/13666 (4.5)	172/1362 (12.6)	570/7057 (8.1)	482/4937 (9.8)
**Drinking water**				
Borehole	9925/16577 (59.9)	113/3401 (3.3)	2457/9498 (25.9)	1476/5877 (25.1)
Piped water	3657/16577 (22.1)	2693/3401 (79.2)	2099/9498 (22.1)	2054/5877 (35.0)
Surface/rain/pond/lake/tank	960/16577 (5.8)	325/3401 (9.6)	1388/9498 (14.6)	670/5877 (11.4)
Well	1897/16577 (11.4)	94/3401 (2.8)	3326/9498 (35.0)	1411/5877 (24.0)
Other	138/16577 (0.8)	176/3401 (5.2)	228/9498 (2.4)	266/5877 (4.5)
**Birth order number**				
1 child	4185/16577 (25.2)	1229/3401 (36.1)	2370/9498 (24.9)	1613/5877 (27.4)
2−3 children	6153/16577 (37.1)	1682/3401 (49.5)	3228/9498 (34.0)	2692/5877 (45.8)
4−6 children	4889/16577 (29.5)	439/3401 (12.9)	2736/9498 (28.8)	1383/5877 (23.5)
7+ children	1350/16577 (8.1)	51/3401 (1.5)	1164/9498 (12.3)	189/5877 (3.2)
**Births in past 5 years**				
1 child	9628/16577 (58.1)	2479/3401 (72.9)	4864/9498 (51.2)	3544/5877 (60.3)
2 children	6322/16577 (38.1)	853/3401 (25.1)	4033/9498 (42.5)	2066/5877 (35.2)
3 children	585/16577 (3.5)	63/3401 (1.9)	567/9498 (6.0)	255/5877 (4.3)
4+ children	42/16577 (0.3)	6/3401 (0.2)	34/9498 (0.4)	12/5877 (0.2)
**Total number of children born**				
1 child	3095/16577 (18.7)	1025/3401 (30.1)	1731/9498 (18.2)	1194/5877 (20.3)
2 children	3678/16577 (22.2)	1113/3401 (32.7)	1879/9498 (19.8)	1597/5877 (27.2)
3 children	2949/16577 (17.8)	694/3401 (20.4)	1557/9498 (16.4)	1273/5877 (21.7)
4+ children	6855/16577 (41.4)	569/3401 (16.7)	4331/9498 (45.6)	1813/5877 (30.8)
**Current breastfeeding**				
No	7971/16577 (48.1)	2512/3401 (73.9)	4846/9498 (51.0)	3492/5877 (59.4)
Yes	8606/16577 (51.9)	889/3401 (26.1)	4652/9498 (49.0)	2385/5877 (40.6)
**Current pregnant**				
No	15515/16577 (93.6)	3300/3401 (97.0)	8671/9498 (91.3)	5543/5877 (94.3)
Yes	1062/16577 (6.4)	101/3401 (3.0)	827/9498 (8.7)	334/5877 (5.7)
**Postpartum amenorrheic**				
No	11580/16577 (69.9)	2713/3401 (79.8)	6694/9498 (70.5)	4286/5877 (72.9)
Yes	4997/16577 (30.1)	688/3401 (20.2)	2804/9498 (29.5)	1591/5877 (27.1)
**Delivery care**				
No	13081/16577 (78.9)	2385/3401 (70.1)	8856/9498 (93.2)	5130/5877 (87.3)
Yes	3496/16577 (21.1)	1016/3401 (29.9)	642/9498 (6.8)	747/5877 (12.7)
**Number of living children**				
1 child	3410/16524 (20.6)	1085/3387 (32.0)	1876/9464 (19.8)	1350/5865 (23.0)
2 children	3837/16524 (23.2)	1136/3387 (33.5)	1981/9464 (20.9)	1709/5865 (29.1)
3 children	3159/16524 (19.1)	683/3387 (20.2)	1609/9464 (17.0)	1262/5865 (21.5)
4+ children	6118/16524 (37.0)	483/3387 (14.3)	3998/9464 (42.2)	1544/5865 (26.3)
**Religion**				
Catholic	2196/16577 (13.2)	0/3401 (0.0)	7806/9498 (82.2)	280/5877 (4.8)
Muslim	83/16577 (0.5)	0/3401 (0.0)	0/9498 (0.0)	16/5877 (0.3)
Other Christians	14273/16577 (86.1)	0/3401 (0.0)	1606/9498 (16.9)	5233/5877 (89.1)
Others	25/16577 (0.2)	3401/3401 (100.0)	86/9498 (0.9)	348/5877 (5.9)
**Mother’s current marital status**				
Never in union	574/16577 (3.5)	1839/3401 (54.1)	1082/9498 (11.4)	304/5877 (5.2)
Currently in union/living with a man	13841/16577 (83.5)	1410/3401 (41.5)	7349/9498 (77.4)	4995/5877 (85.0)
Formerly in union/living with a man	2162/16577 (13.0)	152/3401 (4.5)	1067/9498 (11.2)	578/5877 (9.8)
**Wealth status**				
Poorest	3733/16577 (22.5)	813/3401 (23.9)	2676/9498 (28.2)	1191/5877 (20.3)
Poorer	3589/16577 (21.7)	844/3401 (24.8)	2299/9498 (24.2)	1023/5877 (17.4)
Middle	3244/16577 (19.6)	784/3401 (23.0)	1869/9498 (19.7)	918/5877 (15.6)
Richer	3059/16577 (18.5)	613/3401 (18.0)	1418/9498 (14.9)	1536/5877 (26.1)
Richest	2952/16577 (17.8)	347/3401 (10.2)	1236/9498 (13.0)	1209/5877 (20.6)

## 3 Results

Across the four countries, child survival was high: 98.0% in Malawi, 98.0% in South Africa, 98.8% in Zambia, and 97.2% in Zimbabwe. Sex distribution was balanced, with male children ranging from 49.1% to 52.0%. Most mothers in Malawi (69.4%) and Zambia (69.3%) gave birth between ages 15–20, compared with 46.2% in South Africa and 56.5% in Zimbabwe. Maternal education varied substantially: 69.4% of mothers in Malawi had no formal education, versus 7.1% in South Africa, 47.3% in Zambia, and 12.9% in Zimbabwe. Access to safe drinking water also differed, with borehole water most common in Malawi (59.9%) and piped water predominating in South Africa (79.2%). Current breastfeeding ranged from 26.1% in South Africa to 52.0% in Malawi. These variations in demographic, educational, and household factors may influence under-five survival across countries (Table 2).

**Fig. 3:**
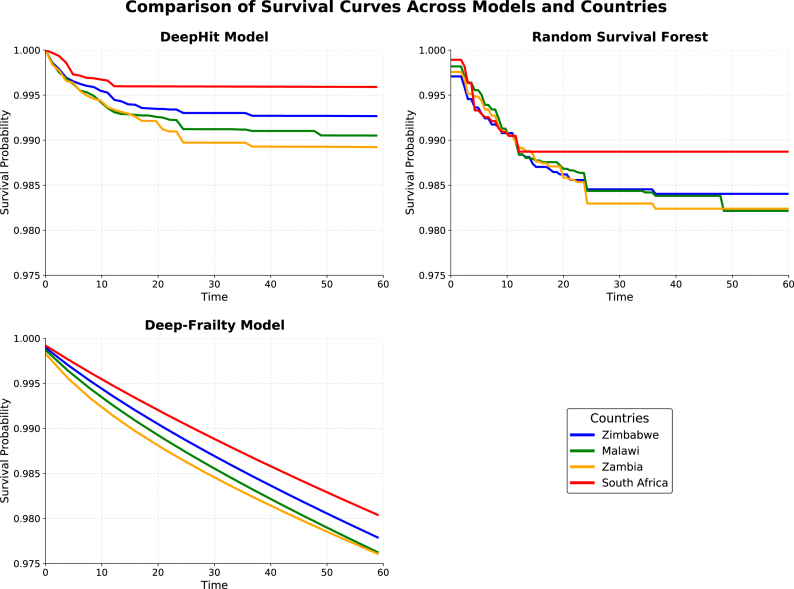
Survival curves for the children under the age of five in Zimbabwe, Malawi, Zambia and South Africa, respectively, extracted from the random survival forest, DeepHit and the DeepFrailty model.

Fig. 3 shows the survival curves from the three models across Zimbabwe, Malawi, Zambia, and South Africa. South Africa consistently exhibited the highest survival probabilities across all three models, whereas Zambia generally showed the lowest survival probabilities over the follow-up period. Zimbabwe and Malawi showed intermediate survival patterns, with Zimbabwe typically demonstrating slightly higher survival probabilities than Malawi. Among the three modelling approaches, the DeepFrailty model produced greater visual separation between countries, with survival curves diverging more distinctly over time. In contrast, the DeepHit and RSF models produced curves that remained relatively closer together during the early follow-up period. Overall, survival probabilities remained high across all four countries throughout the observation period.

**Fig. 4:**
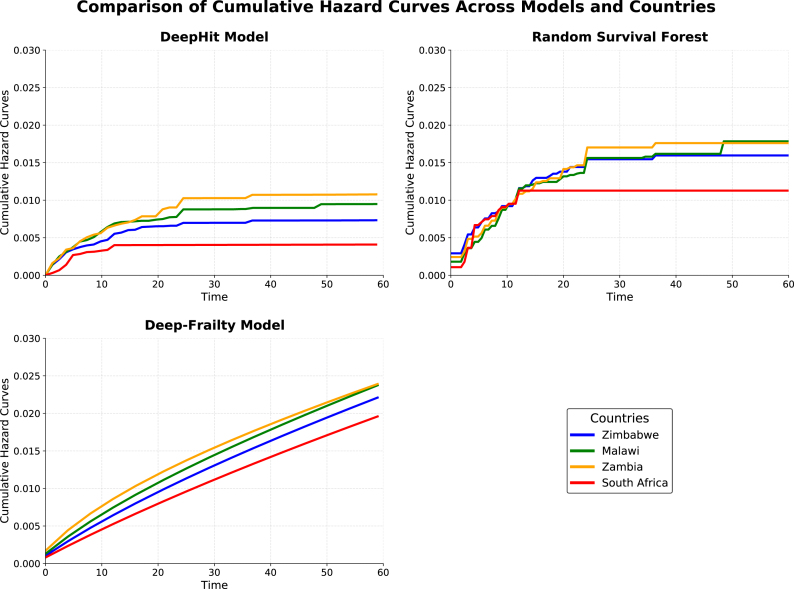
Hazard curves for the children under the age of five in Zimbabwe, Malawi, Zambia and South Africa, respectively, extracted from the random survival forest, DeepHit and the DeepFrailty model.

Fig. 4 shows the cumulative hazard curves across Zimbabwe, Malawi, Zambia, and South Africa for the three modelling approaches. South Africa consistently exhibited the lowest cumulative hazard levels across all models, whereas Zambia generally showed the highest cumulative hazard levels over the follow-up period. Zimbabwe and Malawi showed intermediate hazard patterns, with Zimbabwe typically demonstrating slightly lower cumulative hazard levels than Malawi. Among the three modelling approaches, the DeepFrailty model produced greater visual separation between countries, with hazard curves diverging more distinctly over time. In contrast, the DeepHit and RSF models produced curves that remained relatively closer together during the early follow-up period. Overall, cumulative hazard levels remained relatively low across all four countries throughout the observation period.

**Fig. 5:**
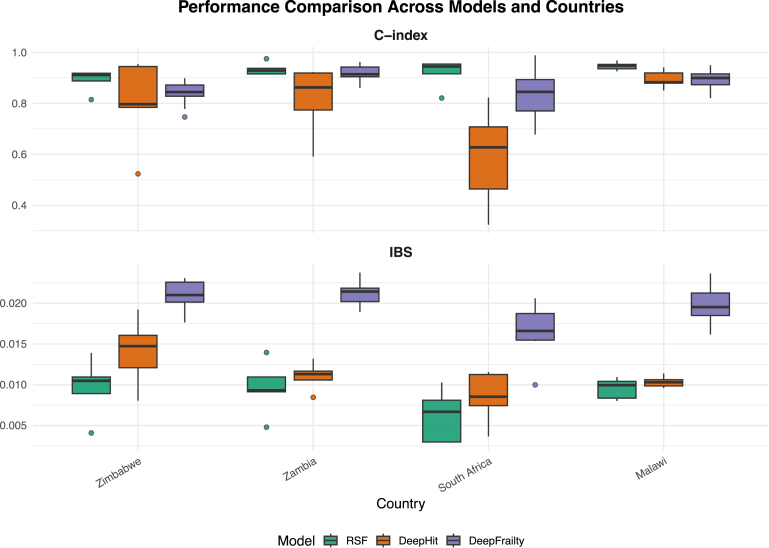
Comparison of concordance across random survival forest (random survival forest), DeepHit, and DeepFrailty models for Zimbabwe, Malawi, Zambia, and South Africa.

Fig. 5 presents the comparative performance of the RSF, DeepHit, and DeepFrailty models for predicting under-five mortality across Zimbabwe, Malawi, Zambia, and South Africa using boxplots of the concordance index (C-index) and Integrated Brier Score (IBS). Overall, the RSF model generally exhibited higher C-index values and lower IBS values across countries relative to the other models. For the DeepHit model, the concordance index was $0.8003\;\left[95\text{\%}\;\text{CI:}\;0.5840,\;1.0000\right]$ in Zimbabwe, $0.8943\;\left[95\text{\%}\;\text{CI:}\;0.8502,\;0.9383\right]$ in Malawi, $0.8135\;\left[95\text{\%}\;\text{CI:}\right)\left(0.6418,\;0.9853\right]$ in Zambia, and $0.5889\;\left[95\text{\%}\;\text{CI:}\;0.3437,\;0.8341\right]$ in South Africa. For the RSF model, the concordance index was $0.8890\left[95\text{\%}\;\text{CI:}\;0.8352,\;0.9444\right]$ in Zimbabwe, $0.9458\;\left[95\text{\%}\;\text{CI:}\;0.9255,\;0.9661\right]$ in Malawi, $0.9336\;\left[95\text{\%}\;\text{CI:}\;0.9027,\;0.9646\right]$ in Zambia, and $0.9177\left[95\text{\%}\;\text{CI:}\;0.8475,\;0.9879\right]$ in South Africa. For the DeepFrailty model, the concordance index was $0.8417\;\left[95\text{\%}\;\text{CI:}\;0.8260,\;0.8574\right]$ in Zimbabwe, $0.8937\;\left[95\text{\%}\;\text{CI:}\;0.8799,\;0.9075\right]$ in Malawi, $0.9159\;\left[95\text{\%}\;\text{CI:}\;0.9035,\right)\left(0.9284\right]$ in Zambia, and $0.8385\;\left[95\text{\%}\;\text{CI:}\;0.8039,\;0.8730\right]$ in South Africa. Across countries, the RSF model generally showed higher C-index values, whereas DeepFrailty showed intermediate performance between RSF and DeepHit. In Zambia, the DeepFrailty model produced C-index values comparable to those of the RSF model. Fig. 5 further compares model performance using the Integrated Brier Score (IBS), where lower values indicate better prediction accuracy over time. For the DeepHit model, the mean Brier score at was $0.0140\;\left[95\text{\%}\;\text{CI:}0.0088,\;0.0193\right]$ in Zimbabwe, $0.0104\;\left[95\text{\%}\;\text{CI:}0.0095,\;0.0112\right]$ in Malawi, $0.0110\;\left[95\text{\%}\;\text{CI:}\right)\left(0.0089,\;0.0132\right]$ in Zambia, and $0.0085\;\left[95\text{\%}\;\text{CI:}0.0045,\;0.0125\right]$ in South Africa. For the RSF model, the mean Brier score at 30 months was $0.0097\;\left[95\text{\%}\;\text{CI:}0.0052,\;0.0141\right]$ in Zimbabwe, $0.0095\;\left[95\text{\%}\;\text{CI:}0.0079,\;\right)\left(0.0111\right]$ in Malawi, $0.0096\;\left[95\text{\%}\;\text{CI:}0.0055,\;0.0138\right]$ in Zambia, and $0.0062\;\left[95\text{\%}\;\text{CI:}0.0022,\;0.0102\right]$ in South Africa. For the DeepFrailty model, the mean Brier was $0.0209\;\left[95\text{\%}\;\text{CI:}0.0188,\;0.0231\right]$ in Zimbabwe, $0.0198\;\left[95\text{\%}\;\text{CI:}0.0170,\;0.0226\right]$ in Malawi, $0.0212\;\left[95\text{\%}\;\right)\left(\text{CI:}0.0195,\;0.0230\right]$ in Zambia, and $0.0164\;\left[95\text{\%}\;\text{CI:}0.0125,\;0.0203\right]$ in South Africa (see Table 3). Across countries, the RSF model generally showed lower IBS values than DeepFrailty and comparable or slightly lower IBS values than DeepHit. Overall, the performance plots showed variation in discrimination and calibration performance across the three modelling approaches and countries.

**Table 3:** Comparison of Mean C-index and Brier score with 95% confidence intervals across models and countries.

Metric	Model	Country	Mean	95% CI Lower	95% CI Upper
C-index	DeepFrailty	Zimbabwe	0.8417	0.8260	0.8574
		Zambia	0.9159	0.9035	0.9284
		South Africa	0.8385	0.8039	0.8730
		Malawi	0.8937	0.8799	0.9075

	DeepHit	Zimbabwe	0.8003	0.5840	1.0000
		Zambia	0.8135	0.6418	0.9853
		South Africa	0.5889	0.3437	0.8341
		Malawi	0.8943	0.8502	0.9383

	RSF	Zimbabwe	0.8890	0.8352	0.9444
		Zambia	0.9336	0.9027	0.9646
	South Africa	0.9177	0.8475	0.9879
	Malawi	0.9458	0.9255	0.9661

Brier Score	DeepFrailty	Zimbabwe	0.0209	0.0188	0.0231
		Zambia	0.0212	0.0195	0.0229
		South Africa	0.0165	0.0125	0.0203
		Malawi	0.0198	0.0170	0.0226

	DeepHit	Zimbabwe	0.0140	0.0088	0.0193
		Zambia	0.0110	0.0089	0.0132
		South Africa	0.0085	0.0045	0.0125
		Malawi	0.0104	0.0095	0.0112

	RSF	Zimbabwe	0.0097	0.0052	0.0141
		Zambia	0.0096	0.0055	0.0138
	South Africa	0.0062	0.0022	0.0102
	Malawi	0.0095	0.0079	0.0111

**Fig. 6:**
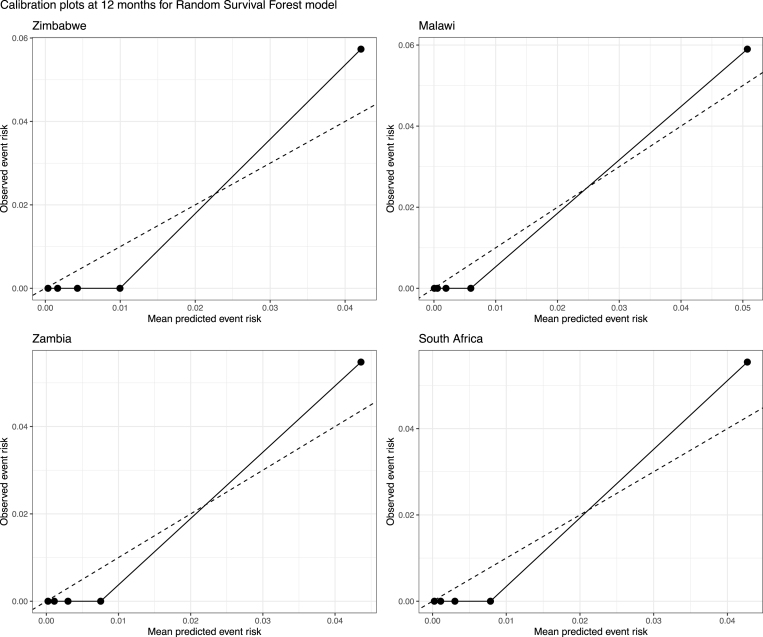
Calibration plots of the Random Survival Forest (RSF) model for predicting under-five mortality at the 12-month prediction horizon in Zimbabwe, Malawi, Zambia, and South Africa.

**Fig. 7:**
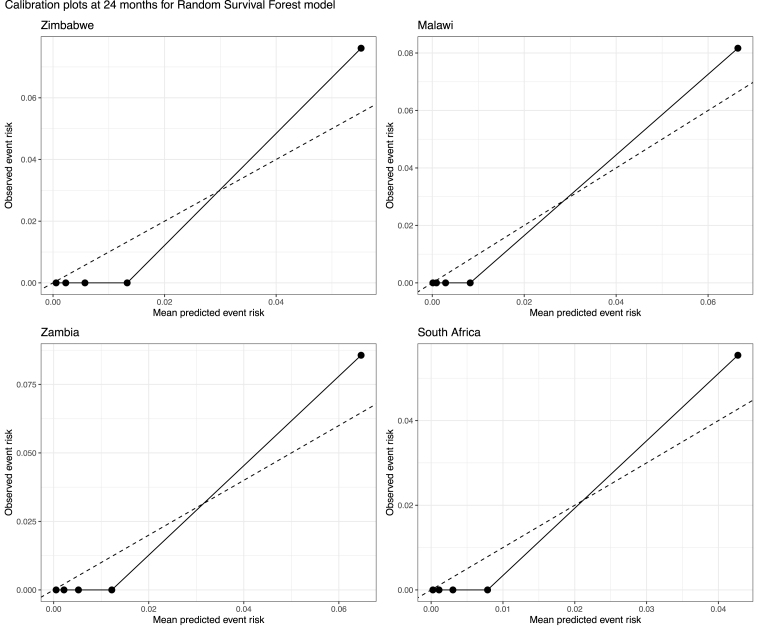
Calibration plots of the Random Survival Forest (RSF) model for predicting under-five mortality at the 24-month prediction horizon in Zimbabwe, Malawi, Zambia, and South Africa.

To further evaluate calibration performance, calibration plots for the RSF model at 12 and 24 months were examined across Zimbabwe, Malawi, Zambia, and South Africa (Fig. 6, Fig. 7). Calibration assesses the agreement between predicted and observed event risks, with curves closer to the 45-degree reference line indicating stronger agreement. Overall, the calibration plots showed that the predicted risks generally aligned with the observed risks across countries at both prediction horizons. At 12 months, the calibration curves closely followed the reference line for most countries, particularly within the lower-risk groups where the majority of observations were concentrated. Some deviations from the reference line were observed at higher predicted risk levels. At 24 months, larger deviations from the reference line became more apparent at higher risk levels across countries, although the calibration patterns remained broadly similar. Malawi and Zambia showed slightly higher observed risks at the upper prediction ranges compared with Zimbabwe and South Africa. South Africa generally exhibited lower overall event risks across both prediction horizons. Overall, the calibration plots demonstrated relatively consistent calibration performance of the RSF model across countries and prediction horizons.

**Fig. 8:**
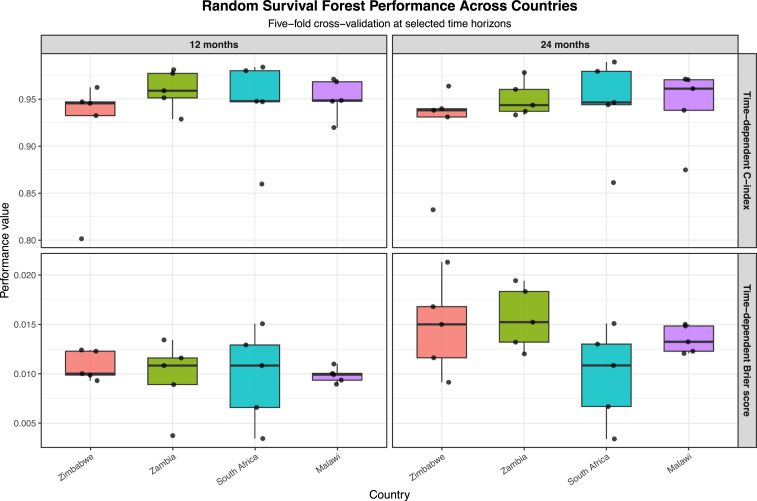
Comparison of Integrated Brier Scores (IBS) and C-Index for the random survival models for Zimbabwe, Malawi, Zambia, and South Africa at two selected time horizons.

To further assess the robustness and stability of the RSF model, five-fold cross-validation was performed at the 12-month and 24-month prediction horizons across Zimbabwe, Zambia, South Africa, and Malawi (Fig. 8). The grouped boxplots summarise the distribution of the time-dependent C-index and time-dependent Brier scores obtained across the validation folds. Across countries and prediction horizons, the time-dependent C-index values were generally high, with most values exceeding 0.90. Zambia, South Africa, and Malawi showed relatively stable C-index distributions across folds, whereas Zimbabwe exhibited comparatively greater variability. The time-dependent Brier scores were generally low across countries at both prediction horizons. At the 12-month horizon, South Africa and Malawi showed lower Brier scores compared with Zimbabwe and Zambia. At 24 months, Brier scores increased across countries, with some increase in variability across folds. South Africa generally showed higher C-index values and lower Brier scores across prediction horizons, whereas Zimbabwe exhibited comparatively wider variability in both metrics. Overall, the cross-validation results demonstrated variation in predictive performance across countries and prediction horizons, while indicating generally stable model performance across validation folds.

**Fig. 9:**
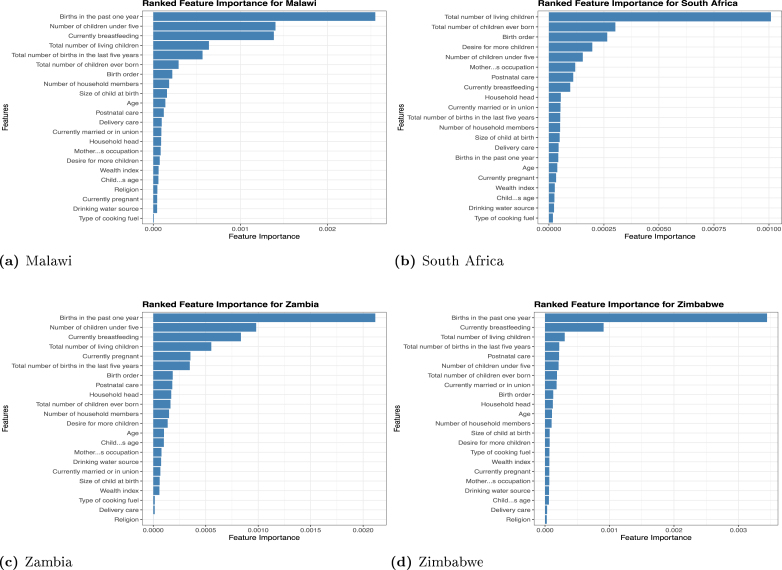
Ranked feature importance for (a) Malawi, (b) South Africa, (c) Zambia, and (d) Zimbabwe.

Fig. 9 shows ranked feature importance for predicting under-five mortality across Zimbabwe, Malawi, Zambia, and South Africa. These rankings are based on PFI [21], which measures the decrease in model performance when each feature is randomly permuted, thus revealing its contribution to the model’s predictive accuracy. Features such as “birth order”, “total number of children ever born”, “total number of births in the last five years”, “currently breastfeeding”, and “births in the past year” ranked among the most important predictors in Zimbabwe. In Zambia, features such as “birth order”, “total number of children ever born”, “total number of births in the last five years”, and “currently breastfeeding” were among the most influential features. For South Africa, “births in the past year” and “total number of births in the last five years” are highly important. “Currently breastfeeding” and “number of children under-five” are also among those that showed relatively high importance. In Malawi, the top features are “birth order”, “total number of children ever born”, “total number of births in the last five years”, “total number of living children”, “currently breastfeeding”, “number of children under-five”, and “births in the past year”.

## 4 Discussion

The results of this study demonstrate consistently high survival probabilities for children under the age of five across Zimbabwe, Malawi, Zambia, and South Africa, with survival estimates remaining above 0.97 across all modelling approaches. These findings align with previous studies reporting improving child survival trends in sub-Saharan Africa [6], [29]. Although overall survival remained favourable, meaningful differences were observed between countries and modelling approaches. South Africa consistently exhibited the highest survival probabilities and lowest cumulative hazard levels, suggesting comparatively better child survival outcomes, whereas Zambia generally showed lower survival probabilities and higher cumulative hazard over time. Zimbabwe and Malawi showed intermediate patterns, with Zimbabwe often performing slightly better than Malawi. These findings likely reflect differences in healthcare access, maternal and child health services, socioeconomic conditions, and broader public health infrastructure across the four countries. The comparative survival and cumulative hazard curves further highlighted important differences between the modelling approaches. While the DeepHit and RSF models produced relatively similar survival trajectories during the early follow-up period, the DeepFrailty model provided noticeably clearer separation between countries. The survival and hazard curves under DeepFrailty diverged more distinctly and continued to separate over time, suggesting that incorporating frailty effects improved the model’s ability to capture latent community-level or cluster-specific variation in mortality risk. This finding emphasises the importance of accounting for unobserved heterogeneity in under-five mortality research, particularly in DHS-type data where important contextual influences such as environmental conditions, healthcare accessibility, cultural practices, and community-level deprivation may not be fully captured by observed covariates. Although the RSF model achieved superior overall predictive accuracy, the DeepFrailty model offered valuable methodological insights by explicitly modelling latent heterogeneity that may influence survival outcomes. The model performance results demonstrated that the RSF model consistently outperformed DeepHit and DeepFrailty across the four countries in both discrimination and calibration performance. RSF achieved the highest concordance index values and the lowest Integrated Brier Scores, indicating superior ability to correctly rank mortality risk while maintaining accurate survival probability estimation over time. The strong predictive performance of RSF was further supported by calibration analyses at 12 and 24 months, in which predicted mortality risks closely aligned with observed outcomes across countries. Although some deviations from the ideal calibration line emerged at higher risk levels, particularly at 24 months, the model maintained generally acceptable calibration performance over longer follow-up periods. The fivefold cross-validation results also demonstrated the RSF model’s strong robustness and stability, with consistently high time-dependent C-index values and low Brier scores across validation folds and countries. These findings suggest that RSF provides reliable and generalisable predictions for under-five mortality across different population settings and prediction horizons. One possible explanation for the superior performance of RSF lies in its ensemble tree-based structure, which is particularly well-suited to handling complex nonlinear relationships, high-dimensional covariate spaces, and sparse epidemiological datasets such as DHS data. Through recursive partitioning and aggregation across multiple trees, RSF can naturally capture hierarchical interactions and latent subgroup structures with relatively limited hyperparameter tuning. In contrast, DeepFrailty and DeepHit rely on neural network optimisation within high-dimensional parameter spaces and may therefore be more sensitive to architectural design, regularisation strategies, and training dynamics. Despite these limitations, DeepFrailty remains an important modelling framework because of its ability to incorporate frailty effects and capture unobserved heterogeneity that traditional machine learning survival models may overlook. Future work may benefit from integrating more interpretable deep learning survival architectures that preserve the flexibility of neural networks while improving transparency and explainability. The feature importance analysis further demonstrated the critical role of household composition, fertility behaviour, and maternal caregiving practices in shaping under-five mortality outcomes across the four countries. Variables such as “birth order”, “total number of children ever born”, “total number of births in the last five years”, “currently breastfeeding”, and “births in the past year” consistently ranked among the most influential predictors. These findings suggest that family structure and maternal reproductive patterns remain major determinants of child survival. Higher birth order and larger family sizes may reflect competition for household resources, reduced parental attention, and increased caregiving burden, all of which may negatively affect child health outcomes. Conversely, breastfeeding consistently emerged as a protective factor, reinforcing extensive evidence regarding its importance in improving immunity, nutrition, and early childhood survival. The feature importance rankings also revealed important country-specific differences that may inform targeted public health interventions. In Zimbabwe and Zambia, fertility-related variables such as birth order and recent birth frequency appeared particularly influential, suggesting that interventions focusing on maternal education, birth spacing, and equitable household resource allocation may help reduce mortality risk. In Malawi, the importance of variables related to family size and the number of living children highlights the potential value of strengthening family planning programmes and maternal healthcare services. In South Africa, breastfeeding and recent birth history were among the most important predictors, suggesting that policies supporting maternal and infant nutrition, breastfeeding promotion, and postpartum care may further improve child survival outcomes. These findings support the need for context-specific interventions tailored to the demographic and household characteristics of individual countries rather than relying solely on uniform regional strategies. Although survival outcomes were generally favourable across all countries, the relatively high overall survival probabilities resulted in limited outcome variability, which may have reduced the discriminative difficulty of the prediction task. This limited variability may partly explain the consistently high performance metrics observed across models. Furthermore, missing covariate information remained an important challenge in this study, as commonly encountered in large-scale epidemiological datasets. Although appropriate preprocessing methods were implemented, future research should consider extending substantive model compatible fully conditional specification multiple imputation approaches to better address missingness in survival modelling settings. Additional future work may also explore incorporating longitudinal covariates, spatial effects, explainable artificial intelligence techniques, and hybrid deep-learning survival frameworks to further improve predictive performance and interpretability in under-five mortality research. Although RSF generally demonstrated superior predictive performance, the observed differences between models should be interpreted with caution, as overlapping confidence intervals alone do not provide a formal test of statistical significance between model estimates. In addition, the relatively low event rate for under-five mortality in the study population may have introduced class imbalance challenges and increased the risk of model overfitting, particularly for more complex deep learning architectures. To mitigate these risks, regularisation procedures, early stopping, and cross-validation strategies were implemented during model training. Nevertheless, future studies with higher event densities or alternative approaches to handling imbalance may further strengthen model stability and generalisability.

## 5 Conclusion

This study demonstrated high estimated survival probabilities among children under the age of five across Zimbabwe, Malawi, Zambia, and South Africa, with survival estimates remaining above 0.97 throughout the follow-up period. Despite these generally favourable patterns, differences in survival and mortality risk were observed across countries, with South Africa consistently showing higher survival probabilities and Zambia exhibiting comparatively lower survival probabilities over time. Variables related to household composition, fertility behaviour, and maternal caregiving practices, including “births in the past year”, “total number of living children”, “total children ever born”, “currently breastfeeding”, and “birth order”, consistently ranked among the most important predictors across countries. These findings highlight the relevance of maternal and household-level characteristics in under-five survival within the analysed DHS populations. Among the three modelling approaches evaluated, the RSF model generally achieved higher discrimination performance, lower prediction error, and more stable calibration patterns across countries and prediction horizons under internal validation. The DeepFrailty model, although showing comparatively lower predictive performance, demonstrated the ability to account for unobserved heterogeneity through the frailty component, allowing incorporation of latent cluster-level variation that may influence survival outcomes. The clearer separation of survival and cumulative hazard patterns observed under the DeepFrailty framework further illustrates the methodological value of incorporating frailty structures in survival analysis. Overall, the findings demonstrate the applicability of machine learning survival models for modelling under-five mortality within the analysed DHS datasets. However, the results should be interpreted in the context of internal validation across the four study populations, and external validation in other settings is required before broader generalisation. Future research may further explore model interpretability, spatial and longitudinal extensions, and improved handling of missing data in survival modelling for child mortality research.

## 6 Limitations

This study has a few limitations. First, survival probabilities were consistently high across all four cohorts, resulting in limited outcome variability, which may reduce the discriminative capacity of the models in identifying subtle differences in mortality risk. Second, the DHS employs a complex survey design involving clustering, stratification, and sampling weights; however, these design features were not explicitly incorporated into the machine learning models. Although the DeepFrailty model accounted for unobserved household-level heterogeneity through a frailty component, the RSF and DeepHit models did not explicitly model clustering effects. Consequently, prediction performance and generalisability should be interpreted cautiously. Third, observations with missing covariate information were excluded from the analysis, which may have introduced selection bias and reduced representativeness if the missingness was not completely random. A formal sensitivity analysis was not performed and is therefore a limitation of the current study. Finally, the analyses were based on internally validated DHS datasets from four Southern African countries, and external validation in other settings is needed before broader application of the models.

## Code availability

The code used for data preprocessing, model development, model evaluation, and figure generation is publicly available at: https://github.com/Justinewits/Deep-Frailty-. The repository includes implementation details, hyperparameter settings, and cross-validation procedures required for reproducibility.

## CRediT authorship contribution statement

**Mafanedza Nephawe:** Writing – review & editing, Writing – original draft, Visualization, Validation, Software, Methodology, Formal analysis, Data curation, Conceptualization. **Wende Clarence Safari:** Writing – review & editing, Writing – original draft, Software, Funding acquisition, Data curation, Conceptualization. **Pierre-Philippe Dechant:** Writing – review & editing, Writing – original draft, Funding acquisition, Conceptualization. **Justine B. Nasejje:** Writing – review & editing, Writing – original draft, Visualization, Validation, Supervision, Methodology, Funding acquisition, Formal analysis, Data curation, Conceptualization.

## Ethics statement

The Demographic and Health Survey (DHS) datasets used in this study are publicly available, de-identified datasets obtained from the DHS Program upon formal request and approval. The DHS surveys were conducted with prior ethical approval from the relevant national Institutional Review Boards (IRBs). The present study used secondary anonymised public-use data and did not involve direct contact with human participants.

Permission to use the datasets for this study titled “DeepFrailty and machine learning modelling for predicting under-five survival in southern Africa” was granted by the DHS Program on 18 May 2026 under the DHS data usage agreement (https://www.dhsprogram.com/data/). All analyses were conducted in accordance with the DHS terms of use and data confidentiality requirements.

Because this study involved secondary analysis of fully de-identified public-use data, additional human subjects ethical review and informed consent procedures were not required at the authors’ institution.

## Funding

This work was supported by 10.13039/100004440Wellcome [321702/Z/24/Z]. The 10.13039/501100000608London Mathematical Society , through the Scheme 5, “Collaborations with Developing Countries” grant [2212], enabling Dr. Justine B Nasejje to travel to LSHTM to collaborate with Dr. Wende C Safari. The funders played no part in the study design, data collection and analysis, decision to publish, or manuscript preparation.

## Declaration of competing interest

The authors declare that they have no known competing financial interests or personal relationships that could have appeared to influence the work reported in this paper.

## Data Availability

The Demographic and Health Survey (DHS) datasets analysed during the current study are publicly available from the DHS Program repository upon request and registration at https://dhsprogram.com/data/available-datasets.cfm.
